# Association between specific domains of the HIC model fidelity scale and the use of coercive measures

**DOI:** 10.1192/j.eurpsy.2026.10937

**Published:** 2026-07-31

**Authors:** H. Demunter, K. Vandenhout, S. Héréus, L. Jansen, C. Kubicek, B. Kutten, C. Scibetta, R. Bruffaerts

**Affiliations:** 1 UPC KU Leuven, Kortenberg; 2 Center for Public Health Psychiatry, KU Leuven; 3 Public Health Psychiatry, WHO Collaborating Center, Leuven, Belgium

## Abstract

**Introduction:**

The development of Psychiatric High and Intensive Care Units (HIC’s) in Belgium, following the Dutch Model (Van Mierlo et al. Werkboek HIC 2013), is an innovative model for patients with acute and severe psychiatric illness. The HIC model aims to provide humane, intensive and need-adapted care with interventions that reduce coercion, focusing on participative processes and continuity of care. HIC implementation processes are supported by a model fidelity scale – *the HIC monitor* - consisting of 10 domains (team structure; team processes; diagnostics, treatment & treatment interventions; organization of care; monitoring; professionalization; act on the protection of persons with a mental illness; healing environment; safety; and evaluation of and feedback on coercion). Although important as an implementation scale, it is not known which specific domains of the HIC monitor are associated with coercive measure use (Van Melle et al. BMC Psychiatry 2020; 20, 469; Bruffaerts et al. Acta Psychiatrica Belgica 2025; 125 (2), 16-26).

**Objectives:**

The objective of the study is to examine the association between specific HIC Monitor subdomain scores and the use of (1) seclusion and (2) mechanical restraint in Belgian HIC-units.

**Methods:**

Between February 2020 and May 2024, 40 audits were organized in all of the 31 HIC units across Belgium to assess the level of implementation of HIC using the HIC Monitor. Associations between monitor subdomain scores and the number of seclusions and mechanical restraints on the ward-level were investigated using a regression model.

**Results:**

First, HIC monitor scores show a strong negative linear association with the number of seclusions (r=0.44; F(1)=5.23; p=.03) but not with the duration of seclusion (r=0.25; F(1)=1.37; p=.26). Higher Monitor scores in 2023 (vs. 2021) were strongly associated with fewer seclusions per bed (r=0.81; F(1)=11.52; p=.02). At the subdomain level, 3 out of 10 domains were significantly associated with a reduction in seclusion frequency: team process (r=0.45), diagnostics, treatment & treatment interventions (r=0.48) and healing environment (r=0.41). Second, HIC monitor scores show no linear association with the number of mechanical restraint (p=0.255). On subdomain level there were no significant associations.

**Conclusions:**

These findings suggest that three domains (team process; diagnostics, treatment & treatment interventions and healing environment) of the HIC monitor scores are associated with reduction in use of coercive measures within psychiatric wards. These domains might serve as valuable tools in the development and improvement processes towards progressively more humane care.

**Disclosure of Interest:**

H. Demunter Employee of: HIC department, UPC KU Leuven, K. Vandenhout Employee of: HIC department, UPC KU Leuven, S. Héréus : None Declared, L. Jansen: None Declared, C. Kubicek: None Declared, B. Kutten: None Declared, C. Scibetta: None Declared, R. Bruffaerts: None Declared

